# Ministerial Indorsements of Humbugs

**Published:** 1895-03

**Authors:** 


### MINISTERIAL INDORSEMENTS OF HUMBUGS.

It is such a common spectacle nowadays to see ministers lending their influence and giving their testimonials to all sorts of patent medicine humbugs, electropoises, etc., that the following from a late issue of the *Christian Advocate* is exceedingly encouraging and refreshing:

"We are sorry to see the names of Christian ministers and persons occupying important positions in the church circulating through the country, indorsing barefaced frauds which are poorly executed, and no better than Indian charms. Whenever a minister of the Gospel allows his name to be attached to anything whose advertisements make statements impossible of fulfillment, or contrary to established principles of science, and especially to be mixed up with all sorts of testimonials written obviously by the most ignorant and uncultivated persons, he loses the respect, and justly, of that class of the community which in the long run fixes the estimate in which he is held.

"When ministers go further and surrender their photographs to patent medicine venders to be used in advertisements and on bottles, they render themselves liable to one of two suspicions: Either that they have an inordinate desire to appear before the public, in which case such extravagant vanity must weaken their influence, or that they have received a compensation for the privilege."

Very well said, indeed! We commend this to certain reverend gentlemen of the cloth in Georgia whose certificates and ornamental features appear constantly among the advertisements of Germetuer, S. S. S., Celery Compound, Oxydonor, etc.

